# Anneau de Keyser Fleischer bilatéral au cours de la maladie de Wilson

**DOI:** 10.11604/pamj.2022.41.44.33122

**Published:** 2022-01-17

**Authors:** Amine Ennejjar, Taha Boutaj

**Affiliations:** 1 Ophthalmology Department “A”, Ibn Sina University Hospital (Hôpital des Spécialités), Mohammed V University, Rabat, Morocco

**Keywords:** Anneau de Keyser Fleischer, cornée, maladie de Wilson, imagerie par résonance magnétique, Keyser Fleischer ring, cornea, Wilson disease, magnetic resonance imaging, MRI

## Abstract

Wilson's disease is a rare autosomal recessive disease resulting in reduced secretion of copper into bile and toxic accumulation of copper in organs, especially in the cornea. Ophthalmological manifestations are important diagnostic criteria. This disease can be adequately treated if diagnosed early. The challenge is to diagnose it in the early stage of liver disease, before it becomes multi-systemic. We here report the case of a 36-year-old patient with a history of Wilson's disease in its three clinical, hepatic, neurological, and psychiatric forms. Ophthalmologic examination showed bilateral Keyser-Fleischer ring.

## Image en médecine

Il s´agit d´un patient de 36 ans, issus d'un mariage consanguin, ayant comme antécédent la maladie de Wilson dans ses trois formes cliniques, hépatique, neurologique et psychiatrique. L´examen ophtalmologique retrouve une acuité visuelle (AV) sans correction chiffrée à 7/10 ODG. Le tonus oculaire était bon. L´examen des annexes retrouve une méibomite. L´examen bio-microscopique à la lampe à fente objective un anneau de Keyser Fleischer bilatéral. La conjonctive comportait des naevus conjonctivaux. La chambre antérieure est de bonne profondeur. Après dilatation, l´examen du cristallin est normal. Le fond d´œil était sans particularités. Le bilan biologique objective un taux sanguin bas de ceruloplasmine à 10 mg/dl et une cuprurie de 24 heures élevée à 126 microgrammes. L'imagerie par résonance magnétique cérébrale du patient révèle un changement de signal généralisé, une gliose et une atrophie des thalamus et du tronc cérébral.

**Figure 1:**
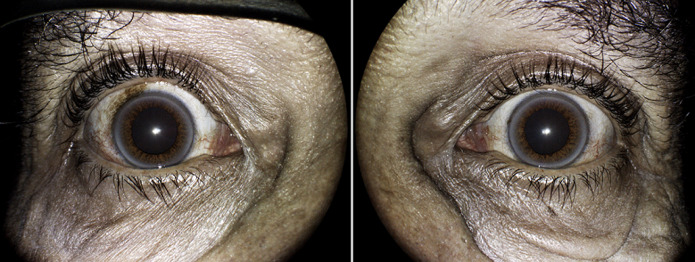
photographie à la lampe à fente; anneau de Keyser Fleischer dans les deux yeux

